# ACKnowledging the role of the Activated-Cdc42 associated kinase (ACK) in regulating protein stability in cancer

**DOI:** 10.1080/21541248.2023.2212573

**Published:** 2023-05-16

**Authors:** Samantha Hodder, Millie Fox, Ana Masara Binti Ahmad Mokhtar, Helen R. Mott, Darerca Owen

**Affiliations:** a Department of Biochemistry, University of Cambridge, Cambridge, UK; b Discovery Sciences, BioPharmaceuticals R&D, AstraZeneca, Cambridge, UK; c Bioprocess Technology Division, School of Industrial Technology, Universiti Sains Malaysia, Penang, Malaysia

**Keywords:** Kinase, cancer, proteostasis, small gtpase

## Abstract

Activated Cdc42-associated kinase (ACK), a non-receptor tyrosine kinase, is an effector for the small GTPase Cdc42. ACK is emerging as an important component of the cancer landscape and thus, a promising target for the treatment of many malignancies. ACK is also being increasingly recognized as a potentially influential player in the regulation of protein homoeostasis. The delicate equilibrium between protein synthesis and protein degradation is crucial for healthy cell function and dysregulation of protein homoeostasis is a common occurrence in human disease. Here, we review the molecular mechanisms by which ACK regulates the stability of diverse cellular proteins (e.g. EGFR, p27, p53, p85 isoforms and RhoGDI-3), some of which rely on the kinase activity of ACK while others, interestingly, do not. Ultimately, further research will be required to bridge our knowledge gaps and determine if ACK regulates the stability of further cellular proteins but collectively, such mechanistic interrogation would contribute to determining whether ACK is a promising target for anti-cancer therapy. In therapeutics, proteasome inhibitors are an efficacious but problematic class of drugs. Targeting other modulators of proteostasis, like ACK, could open novel avenues for intervention.

## Introduction

Collaborative efforts of scientists worldwide have started to piece together the fiendishly complex jigsaw that is cancer. The discovery of oncogenic proteins, pathways and hallmarks has helped resolve portions of the cancer portrait and has aided therapeutic developments. Recent years mark a revolution within cancer therapy as we transition away from traditional, toxic, chemotherapeutic drugs and focus on the development of target-based therapeutics [1]. One challenge associated with effective targeted therapy development remains molecular target identification. Irrespective of a moiety’s potency and/or safety, it will have poor efficacy in the absence of a strong causal link between the disease and the molecular target. Confidence in a selected target within a specific disease scenario grows from genomic analysis and pre-clinical research using representative disease models [2].

The addition and removal of phosphate groups by protein kinases (PKs) and phosphatases, respectively, is a crucial signalling mechanism facilitating the coordination of diverse cellular processes [3,4]. This, together with the fact that kinases are highly susceptible to small-molecule targeting at their active site, has made modulating kinase activity an attractive therapeutic strategy [4,5]. One of the first ‘smart’ drugs, Imatinib, is a tyrosine kinase (TK) inhibitor that inhibits the activity of BCR-ABL [6]. Since its development, Imatinib has revolutionized the treatment of chronic myeloid leukaemia (CML) and has paved the way for kinase-directed drug development [6,7].

**Figure 1.**
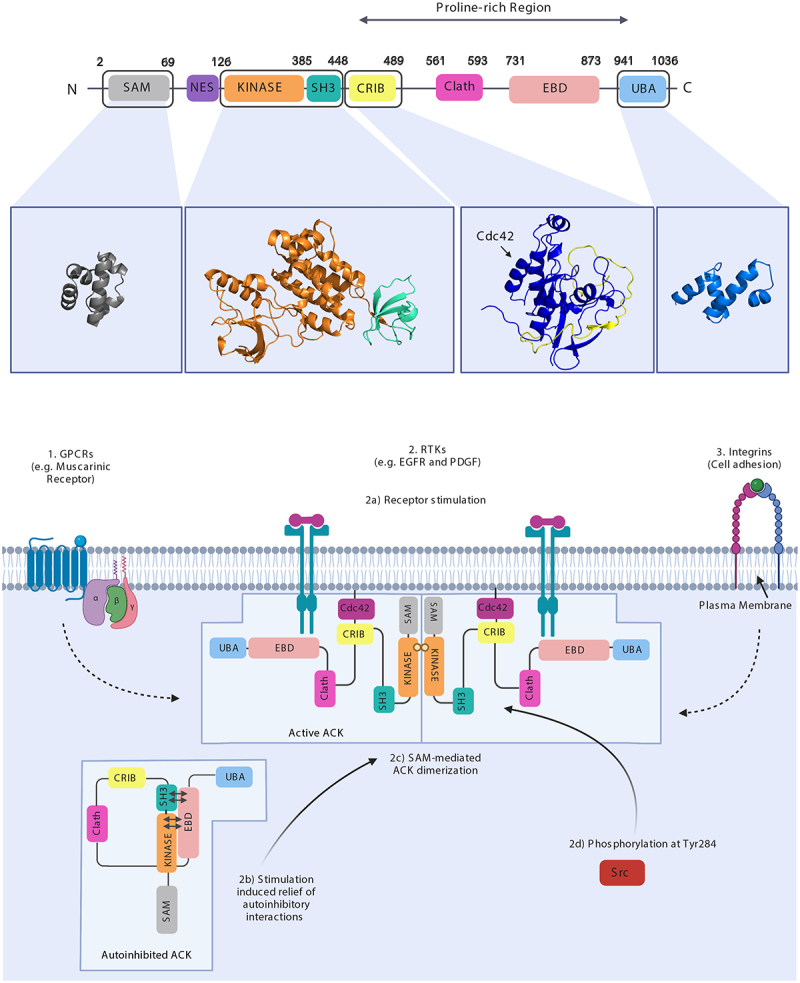
ACK’s architecture and activation. (upper panel) ACK’s domain architecture with domain structures (kinase domain-SH3 PDB: 4HZS, CRIB PDB: 1CF4) or predictions (AlphaFold) shown. ACK encompasses a sterile alpha motif (SAM) domain, nuclear export signal (NES), TK domain, Src homology-3 (SH3) domain, CRIB motif, clathrin binding region, epidermal growth factor receptor-binding (EBD) domain and a ubiquitin association (UBA) domain. Amino acid numbering of domain boundaries is shown above the respective domains. (lower panel) ACK signals downstream of [1] G protein-coupled receptors (GPCRs) [2], Receptor tyrosine kinases (RTKs) and [3] Integrins. RTK stimulation (2a) causes relief of ACK’s autoinhibitory interactions, (2b) SAM-mediated dimerization and (2c) ACK activation. To become fully active, 2(d) ACK is phosphorylated, either by Src or autophosphorylation, on its activation loop at Tyr284. Figure created in BioRender.

Activated Cdc42-associated Kinase (ACK) is a multi-domain, non-receptor tyrosine kinase (NRTK) (Figure 1) [8] and was the first effector protein to be identified for the small G protein Cdc42 [9]. ACK has gathered considerable attention since its identification as a cancer driver [10] and the demonstration that expression of an activated variant promoted the growth of prostate xenograft tumours in mice [11]. Genomic studies have shown ACK to be aberrantly activated in cancer, with gene amplification being the primary change [12]. Horst *et al*. detected *TNK2* amplification and ACK mRNA overexpression in late-stage lung, ovarian, pancreatic, and oesophageal tumours. Of these, ovarian (9%) and lung (14%) cancers displayed the highest alteration frequencies [12]. Similar findings are reported for gastric cancer (GC) where *TNK2* was amplified in 36 (10.7%) of 335 primary GC tumours and is a marker for poor survival [13,14]. These data are corroborated by interrogation of the TCGA database in cBioPortal [8].

In addition, in several cancer types, ACK overexpression and/or activation positively correlate with disease progression and inversely with patient survival [13,15–17]. The levels of activated ACK within prostate cancer biopsies positively correlate with progression to the final castration-resistant prostate cancer (CRPC) stage and the presence of *TNK2* alterations are associated with reduced survival [15,18]. Similar findings were observed in triple negative breast cancer, a particularly aggressive subtype, where ACK mRNA levels in clinical samples also inversely correlate with patient survival [16].

ACK orchestrates its oncogenic activity through interactions with diverse proteins including the androgen receptor (AR) [15,19,20], the oestrogen receptor (ER) [21] and WW domain containing oxidoreductase (Wwox) [11]. Yet, although tremendous progress has been made in recent years, we are still far from having a complete understanding of ACK’s signalling network, which would help determine whether ACK could be an efficacious anti-cancer therapy target. Amongst the more studied cellular roles of ACK in receptor endocytosis and trafficking, regulation of transcription, PI3K pathway activity and regulation of cell motility [8], emerging evidence suggests that ACK also plays a critical role in regulating protein stability. Sustaining the required physiological level of cellular proteins is imperative for healthy cell function and protein imbalances frequently drive cancer development. Thus, with ever-growing evidence incriminating ACK in oncogenesis, it seems appropriate to reflect upon our current knowledge of how ACK regulates protein homoeostasis.

## Activation, regulation and targets of ACK

When surveying ACK’s role in proteostasis, it is pertinent to consider the current understanding of the mechanisms of its own regulation and activation, which will in turn impact on the regulation of proteostasis. ACK (120 kDa, 1038 amino acids) is a NRTK coded by *TNK2* (chromosome 3q29) [8,18]. ACK is comprised of multiple recognized regions, domains and motifs including a sterile alpha motif (SAM) domain, nuclear export signal (NES), TK domain, Src homology-3 (SH3) domain, CRIB motif, clathrin binding region, epidermal growth factor receptor-binding domain (EBD, also known as the MHR, Mig-6-homology region) and a ubiquitin association (UBA) domain (Figure 1) [8]. These domains facilitate functional diversity by enabling interactions with a range of proteins and localization to different cellular compartments e.g. the SAM domain contributes to plasma membrane (PM) localization [18,22]. The position of ACK’s SH3 domain C-terminal to the kinase domain, together with the presence of a CRIB region, makes ACK’s domain architecture unique amongst TKs [23]. ACK also includes several disordered regions, so that its full-length structure is likely to remain unresolved. The structure of several of ACK’s domains have, however, been elucidated or can be reliably predicted (Figure 1) [24–26].

ACK signals downstream of GPCRs [27], integrins [28–31] and RTKs. Stimulation by EGF [31–34], PDGF [31], insulin [17,31], neurotrophins [35], bradykinin [34], Gas6 [11,36] and heregulin [17] all trigger ACK activation (Figure 1). ACK is recruited to RTKs following stimulation both indirectly or directly [8,37]. For example, whereas ACK can interact directly with the EGFR via its EBD, ACK interacts indirectly with the receptors Axl, ALK and LTK through the SH3 domain of the Grb2 adapter protein and proline-rich regions in ACK [36].

Deciphering ACK’s activation mechanism has proven challenging. For most kinases, activation loop phosphorylation is a fundamental step in activation [25,38]. In their inactive conformation, activation loops often obstruct either the ATP or substrate binding site [39]. Phosphorylation relieves this autoinhibition by triggering a conformational change that repositions the loop into its active confirmation [25,38]. Early research showed that ACK undergoes autophosphorylation at Tyr284 but this only increased *in vitro* kinase activity approximately threefold [23,25]. In conventional kinases, activation loop phosphorylation typically results in much greater, > 120-fold, increases [25]. The structures of ACK’s kinase domain in its phosphorylated and unphosphorylated forms showed that ACK adopts an active conformation regardless of its phosphorylation status, similar to other kinases (e.g. the EGFR) which do not require phosphorylation for activation [25]. Further work showed that the SAM domain mediates ACK dimerization, driving autophosphorylation and activation (Figure 1) [22]. The crystal structure of active ACK showed that the kinase domain forms a symmetrical dimer [26], while the importance of dimerization was further highlighted by GST-ACK constructs (which are expected to form stable GST-mediated dimers) showing substantially increased kinase activity [26,40].

ACK is thought to exist in an autoinhibited state, mediated by interactions between the kinase domain and EBD region, and between a proline-rich sequence within the EBD and the SH3 domain (Figure 1) [26,41]. The latter is thought to orientate the EBD to facilitate further interactions with the kinase domain [26]. These analyses have provided insight into ACK’s meticulously choreographed activation mechanism; however, variations on this mechanism have also been proposed. An additional activation hypothesis suggests participation of Src family kinases. Early investigations found ACK to be a substrate of Src and Hck [23] and further work found phosphorylation at Tyr284 by Src family kinases to contribute to ACK activation, adding another potential layer of regulation (Figure 1) [8,42].

Most analysis of ACK to date has concentrated on its role in disease and specifically targets of its kinase activity (see Figure 2 in [8]). For instance, in breast cancer, ACK contributes to tamoxifen resistance. In the presence of tamoxifen, ACK binds to the ER and recruits KMD3A, a histone demethylase, to ER/ACK bound promoters. Subsequently, ACK phosphorylates KDM3A on Tyr114, facilitating KDM3A activation. KDM3A removes repressive mono- and dimethyl marks at H3K9, upregulating ER-dependent gene expression in the presence of tamoxifen [21]. Most notably, the ER-ACK-KDMA3 signalling nexus significantly upregulates expression of homeobox A1 (HOXA1), a potent oncogene also implicated in breast cancer [21].

**Figure 2.**
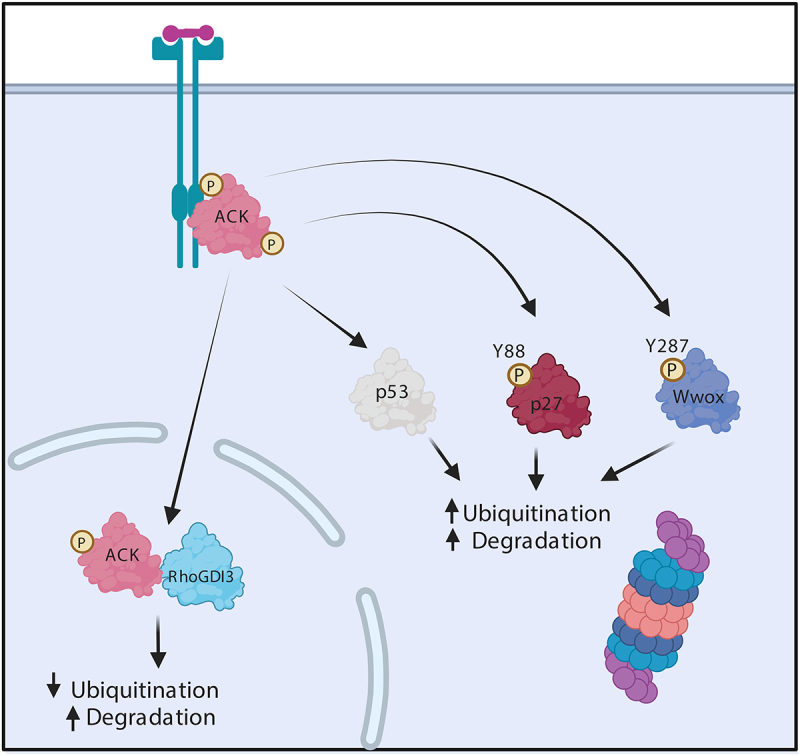
ACK regulates the stability of diverse proteins. Schematic representation of ACK’s involvement in protein homoeostasis. ACK binds p53, p27, Wwox and RhoGDI and phosphorylates all except RhoGDI-3, targeting the proteins for subsequent changes in ubiquitination and proteasomal degradation. Figure created in BioRender.

In castration resistance prostate cancer (CRPC), ACK binds the AR, which facilitates the recruitment of the AR to AR enhancer regions providing an alternative mode of AR activation [19]. ACK phosphorylates the AR at Tyr267 and Tyr363 within the transactivation domain (TAD): mutation of these residues resulted in inhibition of AR target gene expression. As the AR interacts with p160 coactivators via the TAD, it has been hypothesized that ACK promotes AR gene transcription through heightened recruitment of regulatory coactivators and histone acetyl transferases [19].

Several physiological roles for ACK have been established. ACK is involved in the organization of actin polymerization, through phosphorylation and activation of WASP [43] and cortactin [44]. This likely underpins ACK’s role in neuronal extension and branching, possibly linked to its involvement in neurotrophin signalling, where ACK is phosphorylated by the neurotrophin receptor Trk resulting in stimulation of the PI3K and MAPK pathways [35]. ACK is also involved in regulation of T-cell early activation events, via phosphorylation of SLP-76 [45]. Likewise, ACK has been shown to modulate cell survival in *Drosophila*, by interacting with Drk (Grb2) and Yki (Yap) [46]. Interestingly however, despite roles in many fundamental cellular processes, recent research has shown that ACK knockout in mice has no effect on development, behaviour or fertility [47].

## ACK’s role in regulating protein stability

Although research in recent years has enhanced our knowledge regarding ACK’s regulation and signalling functions, less analysis has been conducted on its physiological roles. However, it has become increasingly evident that ACK may play a prominent role in protein homoeostasis.

### Wwox

Wwox (WW domain containing oxidoreductase) is a 46 kDa tumour suppressor protein comprising two N-terminal WW domains and a C-terminal short-chain alcohol dehydrogenase domain. Wwox is found predominantly in hormonally regulated tissues, such as the testis, ovaries, and prostate, where it mediates apoptosis through interactions with partner proteins (e.g. p53 family members) [11]. As described above, ACK is known to play significant roles in cancers involving hormonal regulation [19,21].

Unsurprisingly, Wwox is found to be frequently inactivated in many different types of cancer including invasive breast carcinomas (63%) and aggressive gastric adenocarcinomas (65%). In castration-resistant prostate cancer, ACK promotes cell survival by regulating the stability of Wwox. ACK phosphorylates Wwox at Tyr287, stimulating its polyubiquitination by an unidentified E3 ligase and subsequent proteasomal degradation (Figure 2). Further analysis of prostate cancer biopsies, but not benign lesions, identified elevated levels of activated ACK and reduced levels of Wwox [11]. Similar patterns were observed in a study of hepatocellular carcinoma where pTyr284-ACK levels were shown to positively correlate with tumour grade and inversely with the level of Wwox [48].

### EGFR

**Figure 3.**
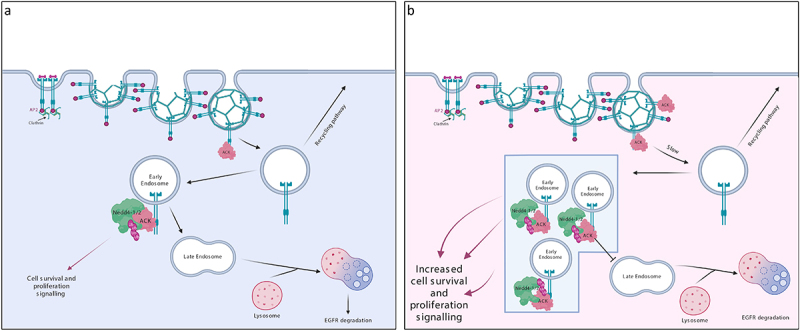
ACK regulates EGFR degradation. (a) in normal physiology, ACK binds to EGFR following EGF stimulation. ACK co-localizes with EGFR during internalization and is subsequently ubiquitinated by Nedd4-1/2 and degraded alongside EGFR. ACK ubiquitination acts as a sorting signal that directs the complex to late endosomes/lysosomes. (b) in cancer, ACK overexpression slows EGFR endocytosis and causes EGFR retention in early endosomes leading to increased cell survival and proliferation signals. Little EGFR is degraded and most internalized EGFR is eventually recycled. Figure created in BioRender.

The EGFR (epidermal growth receptor) is a member of the epidermal growth factor family of receptor tyrosine kinases (ErbBs) that includes EGFR, ErbB2, ErbB3 and ErbB4 [49]. At a cellular level, EGFR signalling mediates cellular growth, survival, proliferation and differentiation [50]. Following EGF stimulation, EGFR monomers undergo dimerization to form asymmetric active dimers, which also form higher order clusters [51–53]. EGFR is then internalized and transported to early endosomes, which either recycle EGFR back to the membrane or fuse with lysosomes resulting in its degradation (Figure 3a) [54–56].

In recent years, ACK has been found to mediate EGFR degradation [33]. After EGF stimulation, ACK binds to the EGFR either directly, through its EBD domain or indirectly, mediated by an adaptor protein (e.g. Grb2) [33,57]. This interaction is dependent on EGFR kinase activity and receptor tyrosine phosphorylation [33]. ACK colocalizes with the EGFR on early endosomes and is degraded alongside EGFR (Figure 3a) [57]. Nedd4-1/2 are the E3 ligases responsible for ACK ubiquitination and lysosomal degradation [57,58]. ACK ubiquitination is therefore thought to act as a sorting signal preventing plasma membrane recycling and directing the ACK-EGFR complex to late endosomes/lysosomes for degradation [57].

In cell studies, ACK overexpression slowed the rate of EGFR internalization (Figure 3b) [59]. This was in agreement with prior studies that showed ACK overexpression limited clathrin-mediated endocytosis of transferrin through ACK-induced clathrin sequestration/redistribution [60]. ACK overexpression was also shown to inhibit EGFR translocation to multivesicular bodies and caused retention of EGFR within early endosomes [59]. As EGFR signalling is not limited to the plasma membrane and can continue after endocytosis (e.g. activation of Ras, Erk1/2 and AKT), such signalling could contribute to cell proliferation and survival [61].

Interestingly, knock down of ACK in HeLa cells has also been shown to impair EGFR degradation, with silencing of ACK resulting in reduced EGFR degradation due to decreased internalization and enhanced recycling of the reduced levels of internalized receptor [59]. In contrast, a more recent study analysed EGFR signalling in ACK KO mice [47]. Although a trend towards increased EGFR levels was observed in keratinocytes from the KO mice, suggesting ACK regulation of EGFR protein levels, this was not significantly different from wt levels. Few significant changes were seen in downstream signalling pathways in normal keratinocytes or in benign skin tumours with the exception of a potential decrease in pAKT levels in ACK KO keratinocytes. Similar results were obtained in ACK KO breast cancer cell lines, although no data from wt lines was available for comparison. This analysis is largely consistent with data from CRISPR and siRNA screens in DepMap public data. Interestingly, some co-dependency can be seen between certain cancers and ACK in siRNA screens but not CRISPR screens, suggesting that longer term adaptation may be occurring: this may also be taking place in KO mice. Further studies of protein levels of the EGFR and other potential ACK targets *in vivo*, along with analysis of disease models featuring mutated or over-expressed ACK would be highly informative in assessing ACK’s regulation of proteostasis.

### The cell cycle regulator, p27

The cell cycle is tightly regulated by the cyclin-dependent kinases (CDKs). The timely inactivation of CDKs is controlled by CDK inhibitors (CKIs) [62]. There are two different CKI families: INK4 (p15, p16, p18, and p19) and CIP/KIP (p21, p27, and p57) [63]. p27 was first described as a Cyclin E/CDK2 complex inhibitor, coordinating cell cycle arrest in G1 [64]. In several types of cancer (e.g. liver, breast and lung), reduced p27 expression is associated with poor prognosis [65]. p27 degradation is coordinated by phosphorylation (at Ser10, Thr157, Thr187, and Thr198). Several protein kinases including c-Src, Yes, Lyn, Brk, JAK2 and Abl have been reported to orchestrate this phosphorylation [66]. Typically, such phosphorylation facilitates the recruitment of the E3 ligase Skp2, which triggers p27 ubiquitin-mediated degradation [67].

Recently, ACK has also been shown to regulate p27 ubiquitin-mediated degradation in head and neck squamous cell carcinoma (HNSCC). ACK levels were found to be significantly higher in HNSCC biopsies in comparison to normal tissue. Subsequent knockdown or inhibition of ACK in HNSCC cell lines (OECM-1, HSC-3, and SAS) enhanced p27 levels and inhibited cell proliferation. Further work uncovered the mechanistic details and ACK was shown to phosphorylate p27 at Tyr88, facilitating the canonical interaction with Skp2 and proteasomal degradation (Figure 2) [66].

ACK is also known to mediate activation of the serine/threonine kinase AKT (see below), a known mediator of p27 degradation [66,68]. Knockdown of AKT in HSNCC cells stably expressing ACK restored the expression of p27, suggesting that AKT also plays an important role in regulating p27’s stability in HSNCC [66]. However, further mechanistic interrogation is required to uncover ACK/AKT’s precise role.

## p53

Alterations to the tumour suppressor, p53, are a common event in cancer, with deletions or mutations found in~50% of cancers and disruption to p53 signalling pathways found in the remaining 50% [69]. p53 acts as a stress-activated transcription factor. In cells not under stress, p53 is ubiquitinated by E3 ligases such as MDM2 and subsequently degraded by the proteasome [70]. One therapeutic avenue under evaluation is the inhibition of MDM2 to restore p53 levels and function [70].

Studies have demonstrated that silencing or overexpression of ACK enhanced or diminished levels of ubiquitination of p53 in gastric cancer (GC) cells, leading to degradation or increased stability of the tumour suppressor (Figure 2) [71,72]. *TNK2* copy number and ACK mRNA levels are significantly elevated in GC in comparison with normal stomach tissues and ACK activation positively correlates with disease progression and inversely with patient survival [71,72]. ACK knockdown in SGC-7901 and MGC-803 GC cells was shown to inhibit proliferation and colony formation due to an induction of G2/M arrest and subsequent cell apoptosis. Similar patterns were observed *in vivo*, where ACK knockdown significantly inhibited tumour growth in mice [71].

Studies to further understand the mechanisms underpinning ACK’s role in cell cycle regulation and apoptosis, analysed protein levels in GC cells after silencing ACK and found 147 with alterations [71,72]. The expression of the cell cycle regulator ECD/hSGT1 was markedly decreased upon ACK silencing and ECD expression in GC positively correlated with ACK expression. Importantly, silencing of ECD also inhibited ACK-induced colony formation, suggesting that ECD acts downstream of ACK in GC cells [71].

Although the full molecular details are not currently available, these data suggest that aberrant levels of ACK-ECD contribute to GC by increasing proteasomal degradation of p53 [71]. Regulation of protein levels of p53 by an ACK signalling pathway is an important observation, with potential application to many disease scenarios.

## The Src-family kinase, CSK

Recent research has outlined a role for ACK in regulating the stability of CSK, a Src-family kinase [73]. The Src-family kinases LCK and CSK play an influential role in T-cell activation. Active LCK triggers T-cell activation but CSK phosphorylates Tyr505, in LCK’s C-terminal tail, facilitating formation of its inactive conformation, therefore negatively regulating T-cell activation [74].

Recent work conducted by Sridaran *et al*. showed that knockdown of ACK in a mouse model resulted in lowered T-cell response thresholds and amplified T-cell responsiveness [73]. Further work added mechanistic clarity, demonstrating that ACK phosphorylates CSK at Tyr18, with knockout of ACK *in vivo* reducing levels of both CSK pTyr18 and LCK pTyr505. ACK KO cells also displayed increased levels of CSK, which prompted an investigation into CSK stability. pTyr18 CSK was shown to be a target for the ubiquitin-proteasome system, indicating that ACK-induced phosphorylation of CSK facilitates the sequential polyubiquitination and degradation of CSK in T-cells. Thus, ACK can both positively and negatively regulate CSK activity, allowing very precise control. This is the first time ACK has been linked to immune system signalling and regulation, and is another example of ACK regulation of proteostasis.

### Class 1a PI3-Kinase p85 isoforms

A yeast-2-hybrid screen was undertaken in our own lab to identify further cellular partners for ACK [75]. This screen revealed ACK binds to Class 1a PI3-Kinase regulatory subunits. As the PI3-Kinase pathway is a major contributor to tumourigenesis, further work was undertaken to investigate ACK’s role within PI3- Kinase signalling [76].

Class Ia PI3-Kinases are heterodimers consisting of a catalytic and a regulatory subunit. In mammals, five regulatory isoforms (p85α, p55α, p50α, p85β or p55γ) and three catalytic isoforms (p110α, β or δ) exist [77]. The C-termini of all five regulatory isoforms comprise two SH2 domains (nSH2 and cSH2) separated by a coiled-coil inter-SH2 region (iSH2), which together form the engagement site for p110 [78].

The regulatory subunits modulate the stability, conformation and localization of the p110 catalytic subunit [78,79]. Under basal conditions, p110 is stabilized in its inactive conformation by inhibitory interactions with the C-terminal region of p85 [76,80]. RTK stimulation triggers heterodimer recruitment to the plasma membrane through interactions between p85’s SH2 domains and RTK phosphotyrosine residues [76]. Such binding alleviates p85’s inhibitory interactions, facilitating the activation of p110’s lipid kinase activity [8,76]. Activated p110 phosphorylates PIP_2_ generating PIP_3_ [8,81], which recruits proteins with pleckstrin homology (PH) domains to the plasma membrane e.g. AKT [81,82]. Following recruitment, AKT is phosphorylated and activated by PDK1 and PDK2 (the major PDK2 activity comes from mTORC2 in the cytoplasm). Active AKT phosphorylates a plethora of downstream substrates that coordinate diverse processes including cell proliferation and survival [83].

**Figure 4.**
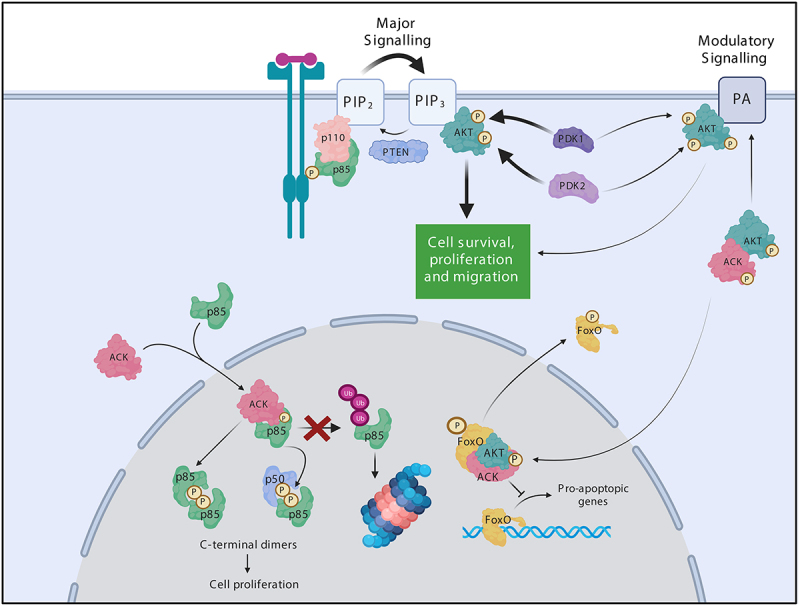
ACK regulates the stability of the PI3-Kinase regulatory subunits. ACK binds and phosphorylates the PI3-Kinase regulatory subunits p85α(Tyr607), p55α(Tyr337), p50α(Tyr307) and p85β(Tyr599). ACK protect the isoforms from ubiquitination and proteasomal degradation. Phosphorylated p85 isoforms dimerize through pTyr607-nSH2 interactions and promote cell proliferation in a PI3-kinase independent mechanism. ACK also regulates a minor pathway in AKT activation. ACK binds and phosphorylates AKT at Tyr176 leading to plasma membrane recruitment via an interaction with phosphotidic acid, after which pTyr176-AKT can be further phosphorylated and activated. pTyr176-AKT in complex with ACK is also found in the nucleus where it acts to phosphorylate Fox0, which is the excluded from the nucleus. Figure created in BioRender.

ACK is emerging as a multifunctional modulator of PI3-Kinase signalling [8]. ACK phosphorylates AKT at Tyr176 leading to its recruitment to the plasma membrane through interactions with phosphatidic acid, where it can be further phosphorylated and activated by PDK1 and PDK2 (Figure 4) [17]. Nuclear translocation of the pTyr176-AKT/ACK complex also leads to activation of AKT, potentially through novel membrane-independent, p53-dependent mechanisms [84] and subsequently results in the phosphorylation of a subgroup of FoxO transcription factors (TFs). FoxO TFs upregulate the expression of genes mediating cell cycle arrest, cell death and DNA repair. ACK-induced FoxO phosphorylation leads to FoxO cytoplasmic translocation, downregulation of FoxO target expression, proapoptotic signalling inhibition and enhanced cancer cell survival [17].

We found that ACK shapes PI3-kinase signalling through an additional node, via the p85 subunits (Figure 4) [75]. ACK binds all p85 isoforms and phosphorylates p85α, p85β, p50α and p55α at an iSH2 domain tyrosine residue (equivalent to Tyr607 p85α) (Figure 4). ACK phosphorylation of p85β promotes cell proliferation in an, as yet undefined, PI3-kinase independent manner. Phosphorylation by ACK was shown to support the formation of p85 dimers, mediated by interactions between the ACK-phosphorylated residue of one monomer and the nSH2 region of the partner isoform. These C-terminal homo/heterodimers reside in the nucleus, which is the primary location for the ACK-p85 interaction (Figure 4) [75]. Previously, N-terminally mediated p85α/p85β dimers (due to BH:BH domain or SH3-PR interactions) have been reported, yet this was the first report of p85 C-terminal dimers. These dimers are of particular interest as they can involve the shorter isoforms p50α and p55α, whose signalling capabilities roles remain less well characterized.

Additionally, ACK mediated phosphorylation also prevents p85 ubiquitination and protects p85 from degradation via the proteasome degradative pathway, resulting in elevated p85 protein levels (Figure 4) [85]. It is tempting to speculate that these phosphorylation-driven elevated p85 protein levels may also contribute to enhanced p85 dimerization.

The stability of p85α is regulated by the tumour suppressor protein p42, the short isoform of ErbB3-binding protein 1. Following binding to p85’s iSH2 domain, p42 chaperones p85 to its E3 ligase (the HSP70/CHIP complex), which promotes protein degradation. Thus, as the iSH2 domain is occluded in pTyr-nSH2 dimers, we hypothesize dimerization itself may also stabilize p85 by preventing its recognition by p42.

### RhoGDIs

The Rho small GTPases (including RhoA, Rac1 and Cdc42) coordinate a number of physiological processes including actin cytoskeletal reorganization, vesicular trafficking and transcriptional regulation [86]. Three different groups of proteins regulate the activity of Rho family members: the guanine nucleotide exchange factors (GEFs), GTPase-activating proteins (GAPs) and guanine nucleotide dissociation inhibitors (GDIs). RhoGDIs bind to the switch regions of small GTPases, maintaining the Rho proteins in their inactive state by preventing nucleotide exchange and to the isoprenyl lipid at the Rho protein C-terminus, physically sequestering the GTPase in the cytoplasm and preventing association with the plasma membrane [86]. However, RhoGDIs also act as chaperones, protecting their targets from degradation and facilitating their delivery to the correct cellular membrane.

Humans have three RhoGDI proteins: RhoGDI-1, RhoGDI-2 and RhoGDI-3. RhoGDI-1 and 2 have particularly high sequence identity (68%). In contrast, RhoGDI-3 has 55–57% sequence similarity with either RhoGDI-1 or 2 and has an extended N-terminus. RhoGDI-1 and 2 have been primarily shown to interact with Rho and Rac subfamily members of Rho family GTPases. However, RhoGDI-3, in addition to having a wider specificity for classical Rho family members, also interacts with atypical small Rho GTPases including Wrch2/RhoV, Rnd2, Miro2 and RhoH [86].

Research in our lab showed that ACK interacts with RhoGDI-1, 2 and 3 [87]. These ACK-RhoGDI protein interactions are not dependent on ACK’s kinase activity and the RhoGDI proteins are not ACK substrates. However, we have found that ACK modulates the stability of RhoGDI-3 (but not RhoGDI-1 or 2) (Figure 2). In contrast to the levels observed when expressing the protein alone, co-expression of ACK and RhoGDI-3 together result in a decrease in RhoGDI-3 levels, most significantly in the nuclear population. This is consistent with the observation that ACK and RhoGDI-3 interact in the nucleus. Interestingly, ACK was found to decrease ubiquitination of RhoGDI-3 but this resulted in degradation of the protein in this case [87]. A decrease in levels of RhoGDI-3 due to ACK could have wide reaching effects given the broad range of its GTPase targets and the multitude of signalling pathways they regulate.

## Concluding remarks

ACK is emerging as an influential modulator of protein stability and homoeostasis. The diverse and precisely choreographed mechanisms by which ACK regulates the stability of several different proteins (e.g. Wwox, p53, p27, the EGFR, p85 isoforms and RhoGDI-3) are starting to be uncovered and likely more will emerge in the near future. Interestingly, although it is clear ACK often modulates protein stability via its kinase activity, this is not true for all its target proteins (e.g. RhoGDI-3). ACK itself contains a UBA domain (Figure 1) and it has been shown that mutation of ACK’s UBA domain enhances ACK stability [88]. Likewise, deletion of ACK’s UBA or SAM domain has been identified to enhance or reduce ACK ubiquitination, respectively, [57].

However, despite our improving understanding of exactly how ACK modulates protein stability, we are far from a complete picture. Further research is required to bridge knowledge gaps and uncover whether ACK modulates the stability of additional cellular proteins, particularly those involved in tumourigenesis or which have tumour suppressor properties.

Ever-growing evidence incriminates ACK in oncogenesis, making ACK a favourable entrant to the cancer drug discovery arena. Accordingly, it is not surprising that several groups have already sought to synthesize ACK-targeting small-molecule inhibitors. Of these, AIM-100 is the most well-studied and has been used extensively *in vitro* to help elucidate ACK’s oncogenic signalling network [89]. Unfortunately, AIM-100 and other ACK inhibitors, lack specificity and possess unfavourable pharmacokinetic properties which have impeded their further development [90]. Using structure-driven medicinal chemistry, Lawrence *et al*. identified (R)-9b [90]. Although (R)-9b has a higher IC50 than AIM-100, it benefits from more favourable pharmacokinetic properties and has recently been shown to have efficacy *in vivo* in castration-resistant prostate cancer tumours [73].

In terms of anti-cancer therapies, proteosome inhibitors are an efficacious part of our arsenal however they come with serious side effects and resistance does develop [91]. Thus, inhibiting ACK could be an efficacious, more targeted approach to successfully modulate protein stability in cancer. Analysis of ACK KO mice also suggest that anti-ACK therapies could be well-tolerated [47]. Ultimately though, a more thorough depiction of ACK’s signalling network and role in regulating proteostasis would give further confidence in ACK as an anti-cancer therapy target and potentially reveal novel avenues for anti-cancer drug discovery.
